# Turning Cellulose Waste Into Electricity: Hydrogen Conversion by a Hydrogenase Electrode

**DOI:** 10.1371/journal.pone.0083004

**Published:** 2013-11-28

**Authors:** Sergey M. Abramov, Elmira R. Sadraddinova, Andrey I. Shestakov, Oleg G. Voronin, Arkadiy A. Karyakin, Nikolay A. Zorin, Alexander I. Netrusov

**Affiliations:** 1 Biological Faculty of M.V. Lomonosov Moscow State University, Moscow, Russia; 2 Chemical Faculty of M.V. Lomonosov Moscow State University, Moscow, Russia; 3 Institute for Basic Problems of Biology RAS, Pushchino, Russia; Louisiana State University and A & M College, United States of America

## Abstract

Hydrogen-producing thermophilic cellulolytic microorganisms were isolated from cow faeces. Rates of cellulose hydrolysis and hydrogen formation were 0.2 mM L^-1^ h^-1^ and 1 mM L^-1^ h^-1^, respectively. An enzymatic fuel cell (EFC) with a hydrogenase anode was used to oxidise hydrogen produced in a microbial bioreactor. The hydrogenase electrode was exposed for 38 days (912 h) to a thermophilic fermentation medium. The hydrogenase activity remaining after continuous operation under load was 73% of the initial value.

## Introduction

Anaerobic decomposition of organic raw materials with thermophilic microorganisms is a promising method for obtaining hydrogen from organic waste. These microorganisms form a microbial consortium that can contain dozens of species. Anaerobic microorganisms that convert complex substrates (e.g., cellulose) into monomer compounds (e.g., cellobiose and glucose) are one of the main functional groups in the consortium [1]. Among them are the following microorganisms: *Clostridium thermocellum*, *Clostridium cellulosi*, *Clostridium thermolacticum*, *Clostridium thermocopriae*, *Caldicellulosiruptor saccharolyticus*, etc. [2]. A second important functional group contains microorganisms that consume monomer compounds and produce hydrogen and carbon dioxide as major metabolites. Prominent members of this group are *Thermoanaerobacterium* spp., *Clostridium* spp., *Thermotoga neapolitana*, *Enterobacter aerogens*, *Thermotoga elfii*, *Caldanaerobacter subterraneus*, etc. [3]. However, molecular hydrogen is not spread in the biosphere because it plays an important role in microbial metabolism. It is continuously produced and consumed by microorganisms that contain hydrogenases. These enzymes catalyse the reversible conversion of hydrogen into protons and electrons [4]. However, it is possible to create selective conditions of cultivation to suppress the hydrogen-consuming microorganisms. In this case, hydrogen will be one of the major final metabolites [5]. At the same time, there are several problems regarding the limited use of hydrogen. The first one is the presence of carbon oxides and sulphide as impurities [6]. The second one is self-inhibition of the hydrogen production [4]. Thus, it is important to remove hydrogen from the bioreactor continuously. Fuel cells could provide the most efficient conversion of hydrogen into electricity. Accordingly, it is likely to immerse hydrogen electrode directly into microbial growth medium to oxidize hydrogen and thus to avoid its inhibitory effect on the metabolism of microorganisms. However, this medium, in the case of heterotrophic microorganisms and mixed substrates, contains a lot of impurities, including hydrolytic enzymes, organic acids, and sulphides [4,7], which can rapidly and irreversibly deactivate the platinum commonly used on electrodes.

The majority of attempts to combine the bioreactor and fuel cell were carried out only with phototrophic bacteria [8]. Previously, we developed hydrogen fuel electrodes based on immobilised hydrogenase [9]. We have shown both their long-term operational stability and their tolerance to major hydrogen impurities, particularly to sulphide. In the previous study, a system combining a bioreactor with hydrogen-producing bacteria and a fuel cell based on hydrogenase electrodes was developed [10]. This system for direct conversion of hydrogen produced by thermophilic cellulolytic microbial consortium into electricity has a maximal power density of 200–250 µW cm^-2^, with 70% activity retained after continuous operation for 72 hours [10]. However, to further develop the system, it is necessary to increase the operational stability parameter. Therefore, this study was devoted to developing the long-term operational stability of a cellulolytic thermophilic microbial bioreactor with an immobilised hydrogenase electrode for continuous electricity production from cellulose wastes.

## Materials and Methods

### Organisms and growth medium

In this study, the consortium of anaerobic heterotrophic hydrogen-producing microorganisms was isolated from fresh cow faeces. Samples of cow faeces were kindly provided by the owner of a private farm in the Moscow region, Russia. A modified Imshenetskij’s medium was used for anaerobic cellulolytic microorganism isolation; it consisted of (in g L^-1^): NaNH_4_PO_4_ – 1.5; K_2_HPO_4_ – 0.5; KH_2_PO_4_ – 0.5; MgSO_4_ x 7H_2_O – 0.4; NaCl – 0.1; MnSO_4_ x 4H_2_O – 0.1 x 10^-2^; FeSO_4_ x 7H_2_O – 0.1 x 10^-2^; CaCO_3_ – 2.0; peptone – 5.0. The pH of the medium was 7.0–7.2 before autoclaving. The following substances were used as organic substrates: paper (filter paper, newsprint or magazine paper, 15.0 g L^-1^), wheat bran (10.0 g L^-1^), wood sawdust (15.0 g L^-1^), kitchen waste (15.0 g L^-1^), straw (15.0 g L^-1^), and different wastes from the brewing industry (yeast, 15.0 g L^-1^ and spent grains, 15.0 g L^-1^). Resazurin was added to the medium at 0.5 mg L^-1^ for confirmation of anaerobic conditions. The medium was prepared anaerobically under 100% argon and autoclaved at 1.0 atm in butyl rubber-stoppered flasks (450 ml volume, with 100 ml of medium in it). 

The structure of the microbial biofilms formed was analysed by scanning electron microscopy (SEM). The samples were prepared according to standard procedure and sputter-coated with a 20-nm thick gold layer. The specimens were examined using a Camscan S2 (Cambridge Instruments, Cambridge, UK) microscope, in secondary electron imaging mode, with a 10-nm optical resolution and an operating voltage of 20 kV. The images were captured using MicroCapture software (SMA, Russia).

Taxonomic composition of the microbial consortium was determined by denaturing gradient gel electrophoresis followed by sequencing techniques. DNA was isolated from enrichment cultures by the standard method [11]. Polymerase chain reaction (PCR) amplification was performed using the system of the universal primers 515 Univ F (5'-GTGBCAGCMGCCGCGGTAA-3') and 907 Bact R (5'-CCGTCAATTCMTTTGAGTTT-3'). The amplicons obtained by PCR were separated on the basis of their melting characteristics on 8% (by volume) polyacrylamide gels with the relative concentration gradient of denaturing agents of 30% to 70%.

### Cultivation of microorganisms

**Figure 1:**
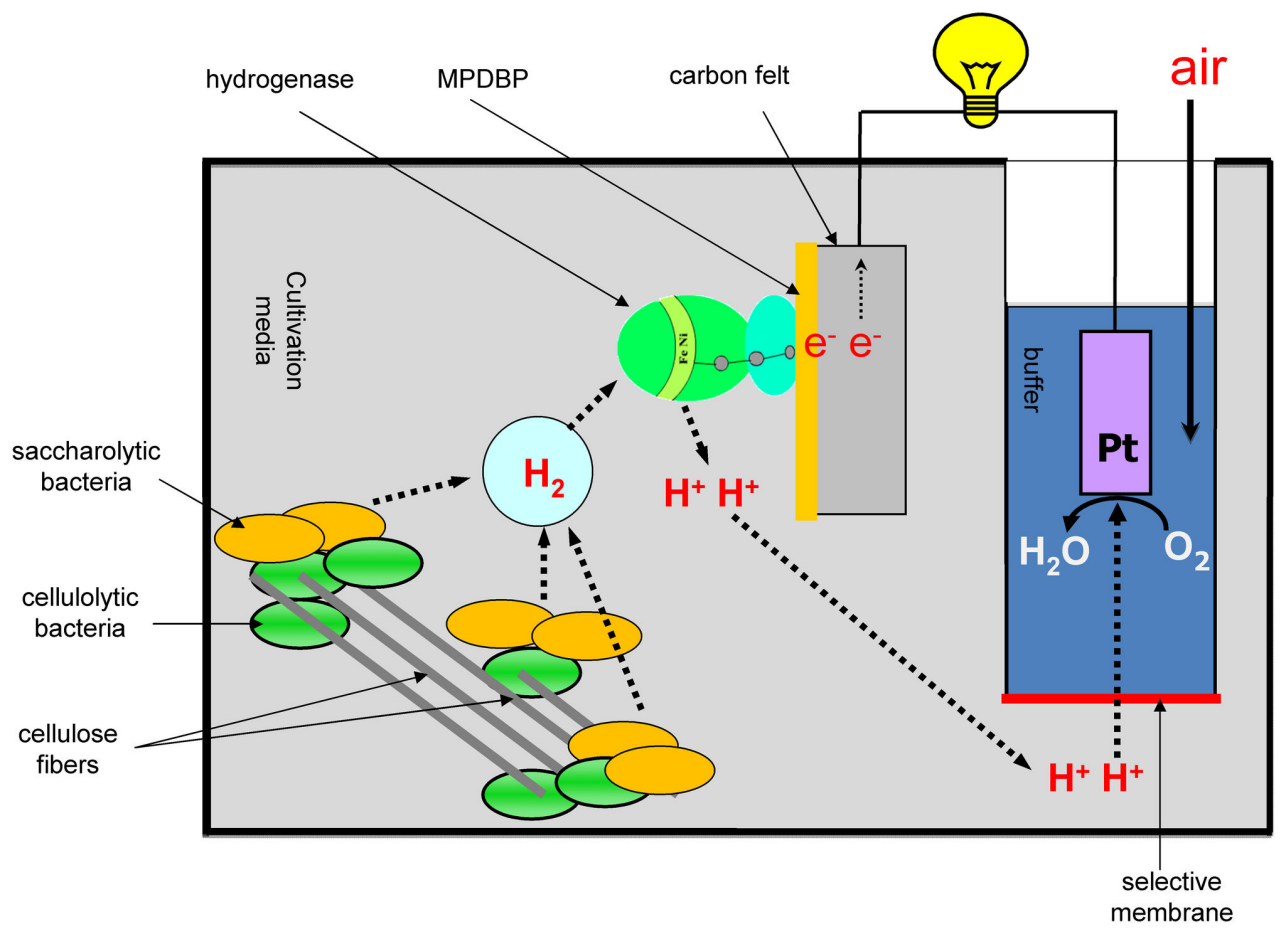
Scheme of the developed bioreactor cell. MPDBP - pyrrole derivative N-methyl-N’-(12-pyrrol-1-yldodecyl)-4,4’-bipyridinium ditetrafluoroborate.

The cultivation of microorganisms was carried out in rubber-stoppered flasks in an automatically controlled bioreactor (BioTron LiFlus GX, South Korea) and in a specially designed bioreactor combined with a fuel cell (the bioreactor cell) at 60 °C. Anaerobic glass bottles were used to study the substrates’ specificity. The cultivation time in the flasks was 7 days (168 h). To set up the bioreactor, a 500 ml flask was used. In this flask, the hydrogen and oxygen electrodes, as well as the system for pressure control were integrated (Fig. 1). Platinum electrodes (platinum deposited on carbon black ‘Vulkan’, 1.7 mg cm^-2^, kindly gifted by the National Research Centre ‘Kurchatov Institute’, Moscow, Russia) were used as a cathode. The cathode chamber was separated from the bacterial medium with Nafion membrane (E. I. du Pont de Nemours and Company, USA). The area of the membrane-separating anode and the cathode chamber was 1.8 cm^2^. The cathode chamber was filled with the phosphate buffer solution (0.05 M KH_2_PO_4_, 0.1 M KCl) adjusted to pH 7. Air was bubbled through the cathode chamber. The volume of the gas phase in the bioreactor cell was 300 ml; the liquid phase was 200 ml. The cultivation time was 912 h (with filter paper as a substrate). The inoculum was added at an amount of 10% by volume of the medium. For cultivation, the bioreactor was placed onto a New Brunswick shaker (35 rpm) and incubated at 60 °C. 

### Determination of metabolic products

The content of the gaseous phase was analysed using the gas chromatograph Crystall 2000.1 (Chromatec, Russia), with a thermal conductivity sensor and a 1000 x 2.5 mm column with activated carbon as the sorbent and argon as the carrier gas; the column thermostat temperature was 150 °C. The analysis of liquid products of cellulose degradation was performed using the liquid chromatograph Crystall 2000.1 (Chromatec, Russia), with a flame ionisation detector, and the microcapillary column ZB-FFAP (Zebron, USA; 15000 mm x 0.32 mm x 0.50 µm). The carrier gas was nitrogen and the flow rate was 15 mL min^-1^. Measurements were carried out under conditions of a temperature gradient in the thermostat from 70 to 160 °C. All chromatograms were processed with the Analytic 2.5 software (Chromatec, Russia). 

### Electrochemical studies

Electrochemical experiments were carried out with Solartron Schlumberger Model 1286 (Solarton, UK), Autolab micro 3 (Metrohm, The Netherlands) potentiostats, and Disco (Motor-Master, Russia) potentiometer. A resistance kit was used to study the generated electric power.

### Electrode preparation

The membrane-bound [NiFe]-hydrogenase from *Thiocapsa roseopersicina* strain BBS was purified according to the procedure described in [12] to 90% of purity and characterised by the activity of 600 mmol (H_2_) min^-1^ mg^-1^ of protein. All solutions throughout this work were prepared using distilled water from a Milli-Q system (Millipore, USA). All the other reagents used were of analytical reagent grade. The hydrogen was produced by water electrolysis (gas supplying system Eldis 15M, Russia). The pyrrole derivative N-methyl-N’-(12-pyrrol-1-yldodecyl)-4,4’-bipyridinium ditetrafluoroborate (MPDBP) was synthesised as described previously [13] and was kindly gifted by Prof. S. Cosnier, Fourier University, Grenoble, France. Carbon felt was used as the electrode material. A solution of MPDBP in CH_3_CN (10 mM) was deposited on carbon electrodes and left to dry for 2 h in ambient conditions. Electrochemical polymerisation of MPDBP was carried out at 0.9 V versus an Ag/AgCl 1 M KCl reference electrode for 10 min in a 0.1 M LiClO_4_ solution (pH 6.0). After the polymerisation, the electrodes were washed with phosphate buffer solution (0.05 M KH_2_PO_4_, 0.1 M KCl, pH 7.0) for 10 min. Then adsorption of enzyme from aqueous solution (1 mg ml^-1^) onto electrodes modified with poly-MPDBP was carried out for 24 h at 4 °C. 

## Results and Discussion

**Figure 2:**
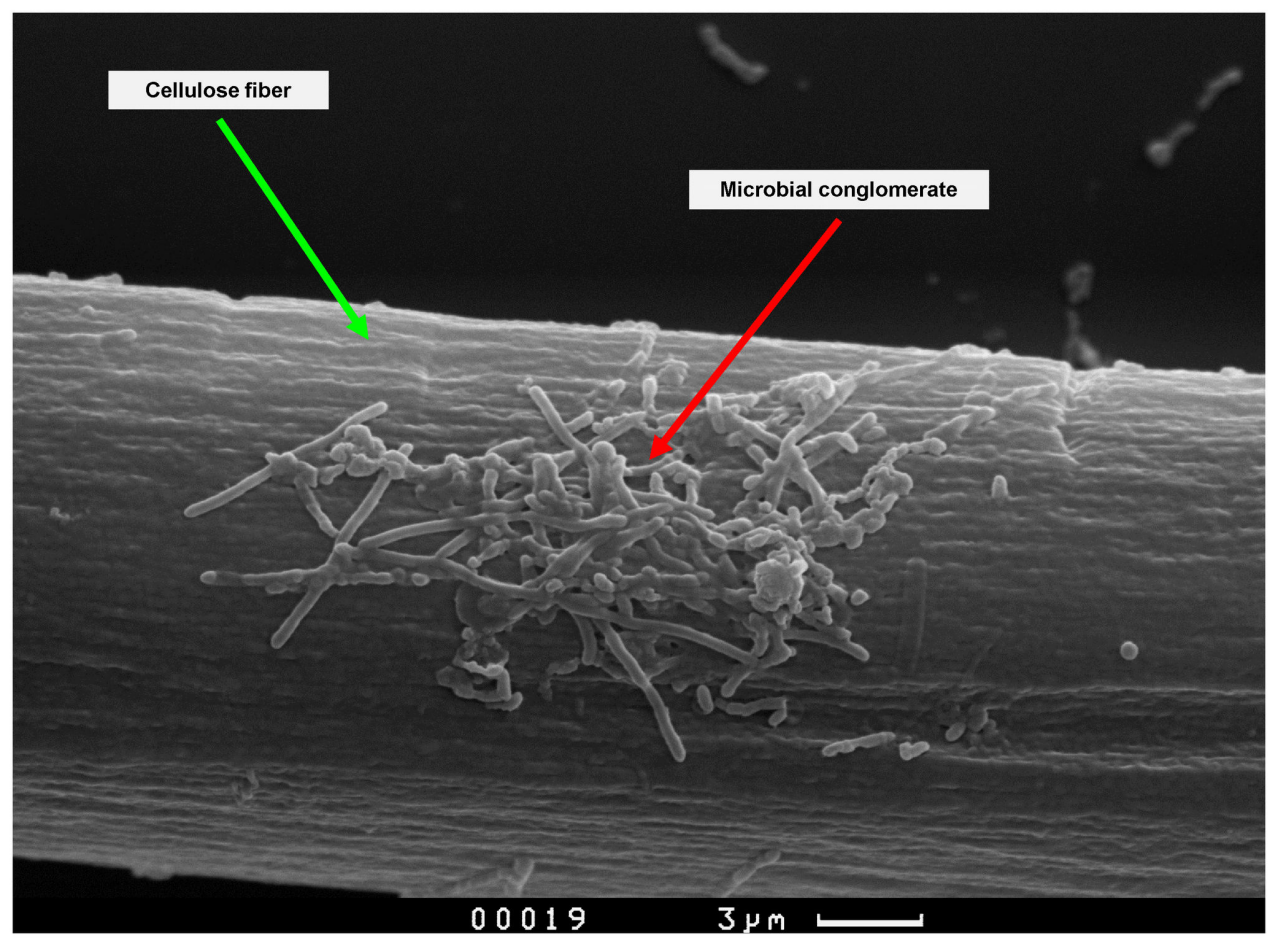
Electron micrograph of the cellulose. The initial stage of cellulose colonisation by isolated microorganisms (3 days of cultivation).

**Figure 3:**
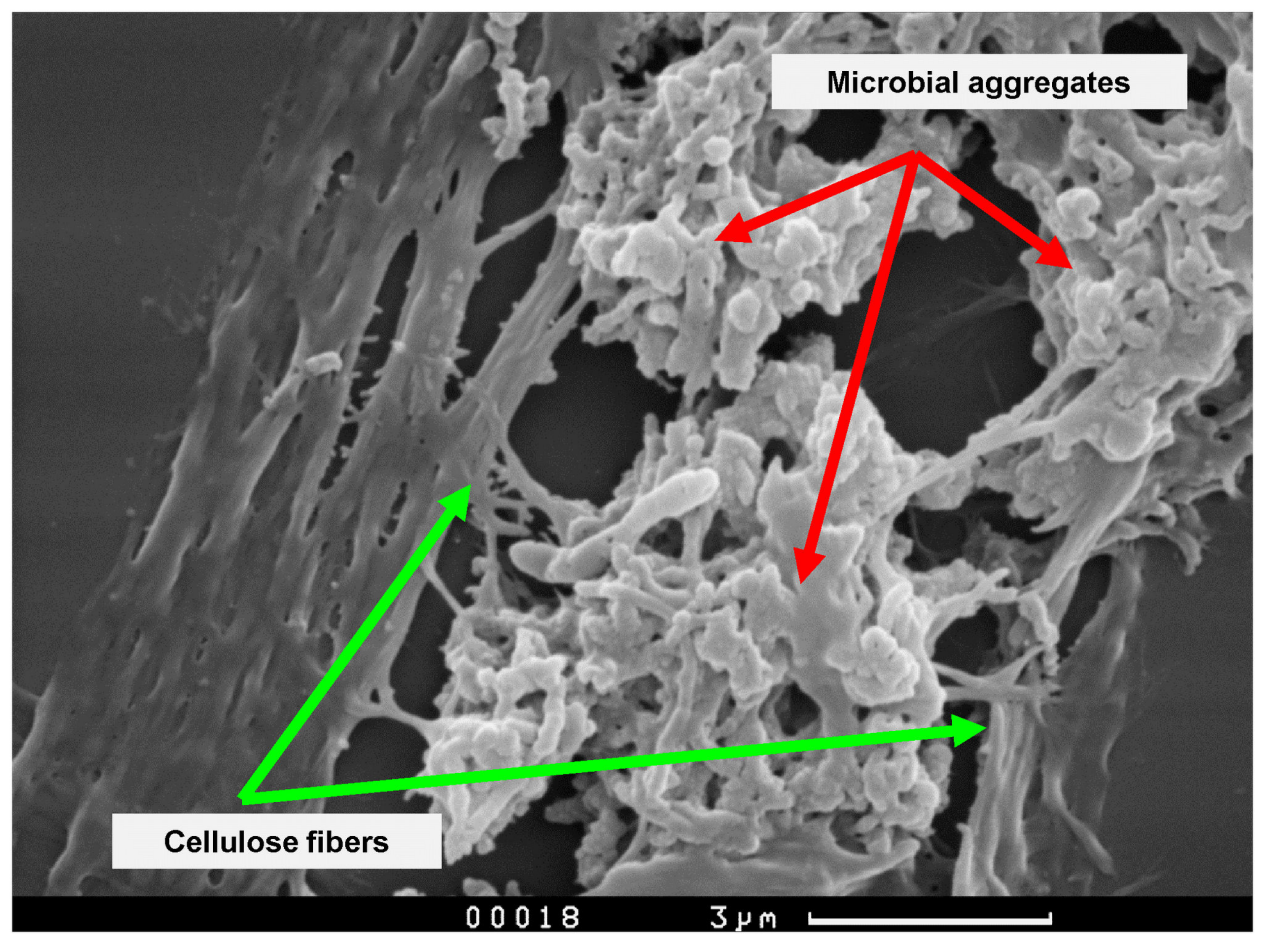
Electron micrograph of the cellulose. **T**he isolated microorganisms immersed in exopolysaccharide matrix on the surface of cellulosic fibers (7 days of cultivation).

The optimum temperature of the hydrogenase isolated from *T*. *roseopersicina* is 80 °С [10]. Therefore, the integration of the hydrogenase electrode in the culture medium of thermophilic microorganisms has to be more attractive than the oxidation of hydrogen produced by mesophilic microorganisms. These microorganisms were isolated from cow faeces. They were characterised by stable growth parameters (dynamics of substrate consumption and accumulation of metabolites), and were cultivated by successive passages, for 2 years at 60 °C, using cellulose (filter paper) as a substrate. The microorganisms formed small clusters of cells around the cellulose fibers. Figures 2 and 3 illustrate the stages of cellulose fiber colonisation. These clusters were immersed in an exopolysaccharide matrix, which promotes a better aggregation of microorganisms on the surface of cellulosic fibers, and closer cooperation between saccharolytic and cellulolytic microorganisms [14]. As the sugars produced during cellulose hydrolysis were consumed immediately by saccharolytic microorganisms, cellulolytic activity was not inhibited by the products of cellulose hydrolysis, since they did not accumulate in the local area where the cellulolytic microorganisms were in contact with the cellulose fiber. Identification of the taxonomic composition of the microbial consortium was carried out. This information allowed us to make an assumption about the distribution of functions within the consortium. The vast majority of the identified microorganisms belonged to the genus *Thermoanaerobacterium* (including *T. thermosaccharolyticum* and *T. aotearoense*). Also, some members of the *Clostridium* genus (including *C. cellulosi* and *C. thermocellum*) were identified. This taxonomic structure is fully consistent with the theoretical concepts of thermophilic cellulolytic consortium composition. It is well known that many members of the *Clostridium* genus have powerful cellulolytic enzyme systems, while species of the genus *Thermoanaerobacter* tend to use monomer sugars as a substrate and are powerful hydrogen producers [2,3]. Different members of both genera are able to grow at high temperatures. The optimum temperature is 55 °C for *C. cellulosi* and 55–60 °C for *Thermoanaerobacter thermosaccharolyticum* [2]. It was shown that a selected microbial consortium was able to hydrolyse 5 g of cellulose (filter paper) per litre of medium for 7 days of cultivation (168 h). An exponential growth phase was characterised by a maximum rate of hydrolysis. However, after 7 days of cultivation, the pH of the culture medium dropped to 4.5–5.0. Consequently, the microbial community ceased to actively hydrolyse cellulose and produce hydrogen. Therefore, we cultivated a microbial consortium at pH stat mode in the bioreactor (BioTron LiFlus GX, South Korea), to study the maximum rate of cellulose decomposition. During the exponential growth phase, the cellulolytic consortium hydrolysed cellulose at a rate of 1.5 g d^-1^ L^-1^ of cultivation medium (0.2 mM L^-1^ h^-1^) and formed up to 1 mM L^-1^ h^-1^ of H_2_. At the end of cultivation, cellulose fibers were not observed in the bioreactor and the culture medium was homogeneous. Moreover, the formation rate of metabolic products (organic acids, H_2_, and CO_2_) was significantly decreased. 

**Figure 4:**
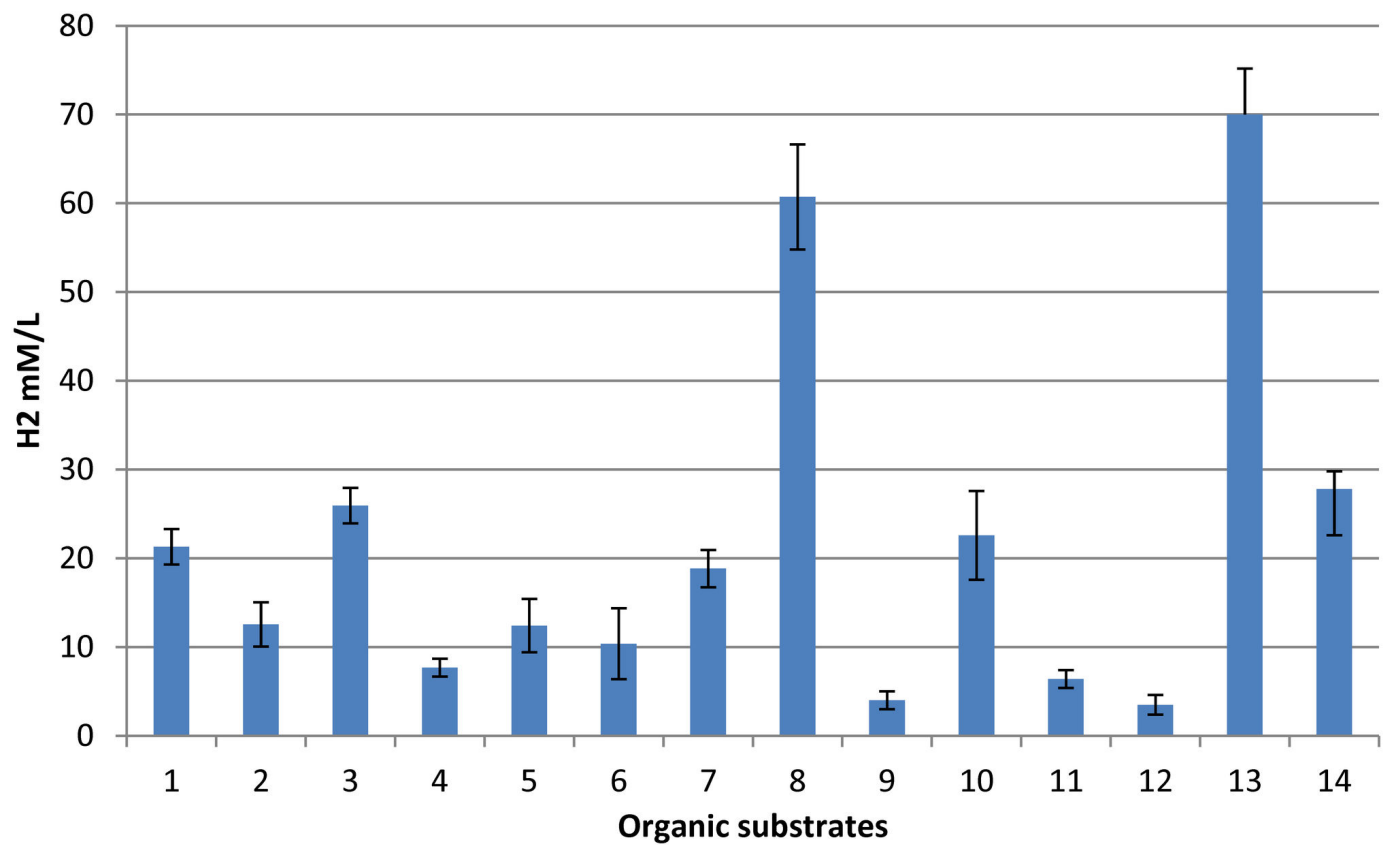
Hydrogen production by the selected microbial consortium using different types of substrates. 1 – glossy white paper; 2 – glossy paper with print; 3 – glossy colour printed paper; 4 – white newsprint paper; 5 – newsprint with print; 6 – colour newsprint paper; 7 – wheat bran; 8 – kitchen waste; 9 – grains; 10 – yeast; 11 – wood sawdust; 12 – straw; 13 – pretreated filter paper; 14 – filter paper. The cultivation time was 7 days.

The possibility of producing hydrogen by microbial processing of different organic wastes in batch mode was studied (Figure 4). The microbial consortium could produce hydrogen during growth not only using digestible food waste, but also during the processing of substrates such as wood sawdust or straw. However, the effectiveness of the process was much lower. Microbial stage efficiency could be increased by pre-treatment of the substrate. The microbial consortium used filter paper that had been pretreated with a solution of 2% sulphuric acid as a substrate, and produced 70 mM of hydrogen for 7 days (168 h) of cultivation. However, in that case, the process of organic waste conversion became more technologically complicated.

**Figure 5:**
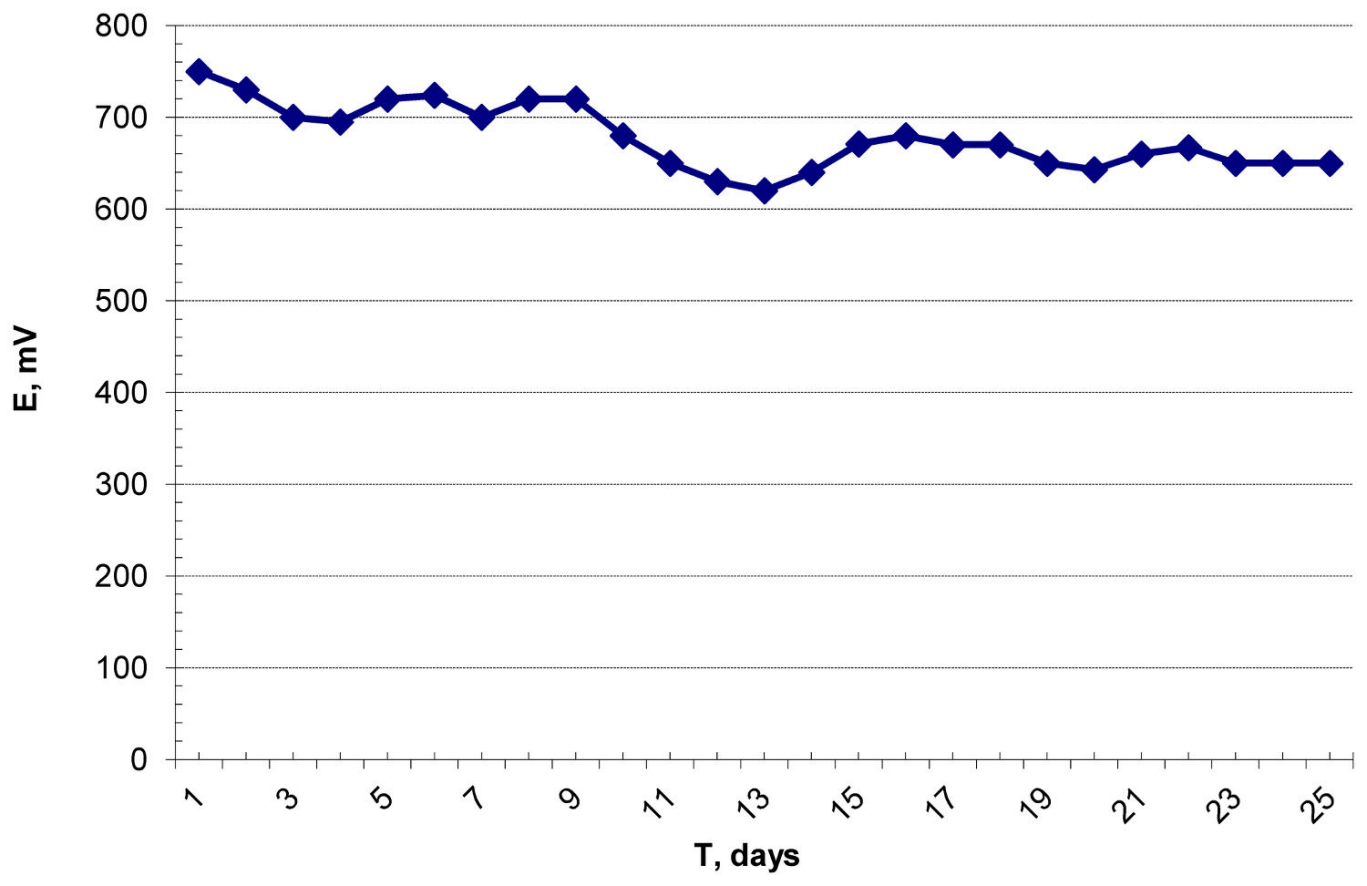
Dynamic of changes in the open circuit potential values. The cultivation time of hydrogen-producing bacteria in the bioreactor cell was 25 days (overpressure in the bioreactor – 0.5 atm, pH 6.7–7.0, 60 °C).

The microbial consortium was cultivated in the bioreactor cell as described previously [10], in a pH stat mode. The pH was maintained between 6.7–7.0. We have previously shown that the pH optimum for hydrogenase is in the range of 6.0–7.0 [15]. Therefore, maintaining the pH in this range helped to keep optimal conditions for the oxidation of hydrogen by hydrogenase. The scheme of the bioreactor cell is illustrated in Figure 1. Study of the enzyme electrode’s stability at a constant load was carried out within 38 days (912 h; Figure 5). The activity of the enzyme immobilised on the electrode was two times lower than the activity of the enzyme in the previous work [10].

**Figure 6:**
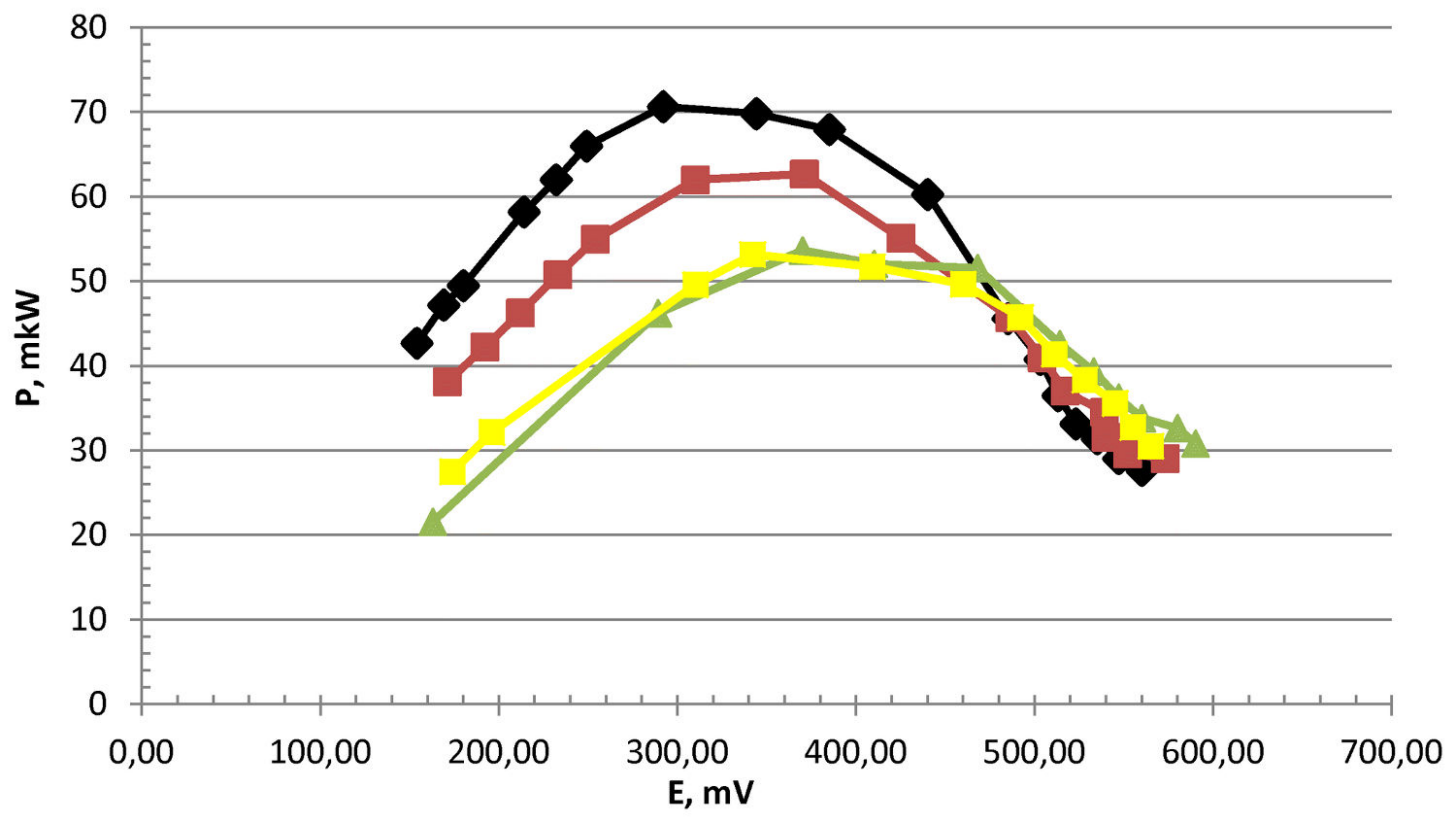
Dependence of power output on overvoltage of the oxygen-hydrogen fuel cell combined with a bioreactor. The cultivation time of hydrogen-producing bacteria in the bioreactor cell was 38 days (25th day – black curve; 29th day – red curve; 34th day – green curve; 38th day – yellow curve, overpressure in the bioreactor – 0.5 atm, pH 6.7–7.0, 60 °C).

The open circuit potential (OCP) was measured for 25 days after the start of cultivation (600 h; Figure 5). A slight drop of OCP from 750 to 650 mV was observed. Such a loss of activity (approx. 14%) correlates well with our previous data for electrodes incubated in microbial media without an electrical load (see 10). After 25 days (600 h) of cultivation, a constant electrical load was added. During the next 13 days (312 h) of cultivation, the power output decreased from 71 to 52 μW (approx. 27% loss of activity; black and yellow curves in Figure 6, respectively). A decrease of any enzyme electrode’s activity can be caused by three main factors: the effect of bacterial proteolytic exo-enzymes, desorption, and the destruction of the redox centre. It is well known that the hydrogenase from *T. roseopersicina* is rather resistant to proteolytic inactivation [16]. The loss of activity due to desorption should be invariable during an experiment, independent of whether the electrode operates with or without a load. Therefore, the key reason for the faster loss of activity during the second part of the experiment (14% after 25 days and 27% after 13 days) was high overvoltage. Such drop of activity in hydrogen oxidation at high overvoltages has been described for all known hydrogenases, even under the strict anaerobic conditions [17]. It is caused by oxidation of the Ni-SI state of the active site to the Ni-B state.

As the effective electrode area of the hydrogenase electrode was not sufficiently large, it was impossible to convert all of the hydrogen into electricity. By increasing the effective surface area of the hydrogenase electrode, we could improve the efficiency of the developed system in terms of conversion of hydrogen into electricity. In the present work, the maximum power density was 71 μW cm^-2^. In most research works, EFCs have a power density in the range of 50–500 µW cm^-2^. The best examples of EFCs have maximum power densities of 1–2 mW cm^-2^ [18]. However, these were achieved with simple carbohydrates used as electron donors in the anode chamber of the EFCs, which are much easier substrates to hydrolyse compared with cellulose [18]. At the same time, the production of hydrogen from cellulose has limitations, depending on the accumulation of hydrogen gas in the culture medium of the bioreactor cell [4]. Therefore, the use of a hydrogenase electrode for direct oxidation of hydrogen in the fermentation medium can increase the production rate of hydrogen and lead to deep cellulose conversion.

## Conclusions

Cellulose is the most abundant glucose-containing polymer produced on Earth annually [19]. Therefore, the conversion of this cheap, renewable, and available substrate into fuel gas can be a very promising source of renewable energy in the near future. The anaerobic conversion of cellulose into renewable fuel is a complex process involving a large number of functional groups of microorganisms. These microorganisms can degrade cellulose to produce hydrogen and short-chain organic acids. This process is environmentally safe and can produce valuable fuel gas (biohydrogen) as well as organic liquids, which can serve as cheap chemical sources for industrial purposes (acetate, butyrate, lactate, ethanol, butanol, iso-propanol, etc.). In all these fermentation processes, hydrogen can be used as a renewable fuel for direct combustion in various engines as well as for fuel cells. Therefore, the production of hydrogen from cellulose, which is a renewable and available source of carbohydrates, is an attractive process for obtaining bioenergy.

Therefore, we isolated a stable cellulolytic hydrogen-producing thermophilic microbial consortium, which hydrolysed cellulose at a rate of 0.2 mM L^-1^ h^-1^ and produced up to 1 mM H_2_ L^-1^ h^-1^. Under similar conditions, the rates of filter paper hydrolysis and hydrogen formation by *Clostridium cellulolyticum* were 0.15 mM L^-1^ h^-1^ and 0.57 mM L^-1^ h^-1^, respectively [2]. It was shown that the enzyme electrode, based on hydrogenases, was able to convert the hydrogen produced by isolated microorganisms into electricity without any additional purification steps. The maximum power output achieved was 71 µW/cm^2^ of hydrogenase electrode. During continuous operation under load, the power value of the electric current decreased. The drop in hydrogenase electrode activity was largely due to the accumulation of microbial metabolites in the culture medium, which were the cause of enzyme inactivation. In addition, changes in environmental conditions during the cultivation of microorganisms can result in the loss of enzyme from the surface of the felt. Perhaps lost enzymes were initially easily available for hydrolysis or badly linked to felt. However, it should be noted that for the 13 days (312 h) of operation under load, the power value of the electric current dropped only by 27%. Further stability of the developed system can be improved by using the novel polymeric compounds for immobilization of the hydrogenase on the surface of electrode [18].

The problem of the EFC’s substrate specificity was solved by using hydrolytic hydrogen-producing microorganisms for substrate hydrolysis. The hydrogenase electrode was stable for at least 38 days (912 h) of incubation in a culture medium of hydrolytic hydrogen-producing microorganisms, capable of processing various kinds of organic waste, as well as cellulose. 
